# Comparison of the Severity of Zoster-Associated Pain and Incidence of Postherpetic Neuralgia in Patients with and without Pre-Existing Spinal Disorders at the Same Spinal Nerve Level: A Retrospective Multicenter Study

**DOI:** 10.3390/jpm13091286

**Published:** 2023-08-22

**Authors:** Ji Seon Chae, Jiwoong Im, Yong Ju Choi, Hyun Jung Lee, Won-Joong Kim

**Affiliations:** Department of Anesthesiology and Pain Medicine, College of Medicine, Ewha Womans University, Seoul 07804, Republic of Korea; chaejitt@hanmail.net (J.S.C.);

**Keywords:** postherpetic neuralgia, herpes zoster, varicella-zoster virus infection, spinal disease

## Abstract

The incidences of herpes zoster (HZ) and postherpetic neuralgia (PHN) are significantly influenced by age. As individuals age, the occurrence of spinal disorders increases, thereby raising the likelihood of HZ and PHN coexistence. Considering this, our study aimed to explore the potential impact of pre-existing spinal disorders at the nerve level where HZ developed, on the severity of zoster-associated pain (ZAP) and the incidence of PHN. For our investigation, we retrospectively analyzed a total of 237 patients who presented with HZ and ZAP at various sensory levels (cervical, thoracic, lumbar, and sacral) with or without pre-existing spinal disorders. The presence or absence of spinal disorders at the sensory level affected by HZ was determined using computed tomography or magnetic resonance imaging. Our study results revealed that the group with spinal disorders at the sensory level where HZ developed did not exhibit an increased incidence of PHN. However, 3–6 months after HZ onset, this same group showed significantly higher ZAP scores compared to the group without spinal disorders. It implies a need for heightened pain management, as the coexistence of these conditions can increase pain severity. This study furnishes an initial standpoint to delve into intricate interactions between two diseases.

## 1. Introduction

Herpes zoster (HZ) is a prevalent condition characterized by a painful, unilateral, vesicular rash arising from the reactivation of a latent varicella-zoster virus (VZV) in the ganglia of the cranial nerve or dorsal root. The most noticeable feature is rash, but the most incapacitating symptom is zoster-related acute and chronic pain [1,2,3].

Despite not being lethal, HZ can cause zoster-associated pain (ZAP), which encompasses acute HZ pain and chronic postherpetic neuralgia (PHN). ZAP results from virus-induced damage and heightened sensitivity of affected segmental sensory neurons. The reactivated virus damages both peripheral and central nerves and triggers inflammation, immune responses, and varying degrees of neuronal loss in the affected spinal ganglia [2,3]. Approximately 8–24% of all patients with HZ experience PHN [1,4,5]. Intense ZAP can considerably restrict a patient’s daily activities and lead to a significant decline in their functional capacity and quality of life [2,3].

PHN is associated with several risk factors, including decreased cell-mediated immunity, advanced age, female sex, presence of a prodromal phase, more severe rash, acute intense pain, insufficient nutrient intake, and psychosocial risk factors [6,7]. The incidence of PHN is significantly influenced by age, with approximately 18% of individuals aged >50 years and 33% of those aged ≥80 years with shingles developing PHN following the onset of the rash [6,8].

The increasing age of the global population is expected to result in a significant increase in the incidence of spinal disorders in the coming decades, thereby posing a substantial global health challenge [9]. With increasing age, the incidence of spinal disorders and HZ, as well as PHN, shows a corresponding increase [10]. Consequently, it is conceivable that the probability of co-occurrence of spinal disorders and HZ, potentially affecting the same spinal nerve level, becomes progressively higher as one ages.

We hypothesized that the coexistence of HZ and spinal disorders at the same nerve root level, as well as the synergistic increase in pain, could also have an impact on the development of PHN since severe pain at the onset of HZ is considered one of the major factors leading to its development. To our knowledge, no studies have investigated the relationship between these two conditions and their potential impact on prognosis, particularly regarding the possible exacerbation of symptoms. Therefore, we examined whether ZAP increases due to a pre-existing spinal disorder and whether the probability of PHN increases thereafter.

## 2. Materials and Methods

### 2.1. Ethics

This study was a retrospective comparative review of chart data, ensuring the preservation of patient privacy and data confidentiality throughout the research process. The institutional review board centers of Ewha Womans University Seoul Hospital (SEUMC 2023-02-014) and Ewha Womans University Mokdong Hospital (EUMC 2023-03-041) approved the study. Due to the absence of direct contact with the study population and the removal of all patient identifiers from the dataset during initial collection, the requirement for written informed consent was waived.

### 2.2. Participants

We included 237 patients with ZAP who received medical treatment at the outpatient pain centers of the Ewha Womans University Seoul Hospital and Mokdong Hospital between 2019 and 2022. Electronic clinical records and survey responses were retrospectively reviewed to confirm adherence to inclusion criteria.

The inclusion criteria were as follows: (1) HZ at the cervical, thoracic, lumbar, or sacral sensory level; (2) ZAP scores ≥4 (evaluated at the first visit using the numeric rating scale (NRS) scores); (3) patients within one month of HZ infection; (4) patients who had undergone magnetic resonance imaging (MRI) or computed tomography (CT) with images that could determine the presence or absence of a spinal disorder; and (5) patients aged ≥18 years.

The exclusion criteria were as follows: (1) patients who had already been diagnosed with HZ in the past several years; (2) patients with no symptoms, even if a spinal disorder was discovered during the imaging examination; (3) patients who received radiofrequency ablation for pain relief related to ZAP or underwent invasive interventional treatments, including surgery, vertebroplasty, or kyphoplasty for pain relief related to spinal disorders; and (4) patients who had diagnostic codes for cancer, human immunodeficiency virus infection, organ transplants, chronic obstructive pulmonary disease, rheumatic diseases, and autoimmune diseases, which are considered potential confounders of HZ and PHN as the patient may be immunosuppressed as a consequence of these conditions or their treatments [11,12,13,14].

### 2.3. Selective Nerve Root Block (SNRB) Procedures

While other invasive therapeutic interventions were excluded from this study, selective nerve root blocks (SNRB) were included as a commonly performed therapeutic intervention for both ZAP and pain associated with spinal disorders [4,15,16].

SNRB procedures were performed under fluoroscopic guidance, with the patients placed in a prone position for thoracic and lumbosacral procedures. Following standard sterile preparation, the fluoroscope was rotated obliquely to visualize the space between the nerve root to be treated and the pedicle on the ipsilateral side. The needle was carefully advanced next to the nerve root and placed slightly inferior to the pedicle. In the lateral image, the final position of the needle was observed just anterior to the zygapophyseal joint, whereas, in the anterior–posterior (AP) view, it was observed just inferior to the pedicle. After ensuring no vascular or epidural uptake, 2 mL of 0.2% ropivacaine containing 5 mg dexamethasone was administered.

The anterolateral approach was used for the cervical procedure, and patients were placed in the supine position. The fluoroscope was angled obliquely, and a needle was advanced towards the medial side of the superior articular process, located just behind the foramen. Once the needle contacted the superior articular process, it was carefully advanced ventromedially towards the posterior section of the foramen. Before administering 1 mL of 0.2% ropivacaine and 5 mg of dexamethasone, negative vascular and epidural uptake was confirmed using a contrast medium. All procedures were performed by skilled pain physicians.

### 2.4. Definition of PHN

The evaluation period was defined as a range of 1–6 months after HZ onset. Numerous research studies have defined clinically significant PHN as persistent pain, with an intensity of ≥3 points on the NRS [10,17,18]. Accordingly, we defined ZAP as any pain related to HZ with an NRS score of ≥1, while PHN was defined as pain with an NRS score of ≥3 that persisted for at least 3 months after HZ infection. Cases with an NRS score of <3 or in which treatment was terminated owing to pain resolution were classified as non-PHN cases.

### 2.5. Confirmation of Spinal Disorder

This study included representative spinal disorders associated with degenerative etiologies [9,19,20,21]. The presence of a spinal disorder was confirmed by two anesthesiologists or based on the results read by a radiologist. In case of disagreement, the patient was excluded from the study.

### 2.6. Outcome Measures

The primary outcome measure for this study was the NRS score for ZAP, which was evaluated at 1–6 months following the onset of HZ. The secondary outcomes included the incidence of PHN, medication dosages, and medication discontinuation rates. The study examined the PHN rate among patients who had ZAP and the entire patient group, which included those who had recovered. If any other anticonvulsants were used, they would need to be explicitly stated. However, in this study, there were no patients who received anticonvulsants other than gabapentin and pregabalin. Therefore, all anticonvulsant doses were converted to the equivalent dose of pregabalin [22,23] and opioids were titrated using the morphine equivalent daily dose (MEDD) [24].

### 2.7. Statistical Analysis

Continuous variable normality was examined using the Shapiro–Wilk test. The continuous variables were presented as mean ± standard deviation or median (interquartile range), while categorical variables were expressed as numbers (percent). The Mann–Whitney U test or independent *t*-test was used to compare the outcomes between the 2 groups for continuous variables, whereas the Chi-square test or Fisher’s exact test was used for categorical variables. At each time point, the NRS scores were compared using repeated-measures analysis of variance (ANOVA) with Bonferroni correction for post hoc comparisons. The discontinuation rate of drugs was assessed by the Kaplan–Meier survival method and differences between both groups were compared using the log-rank test. SPSS version 23.0 (IBM Corp., Armonk, NY, USA) was used for statistical analysis, and a *p* value < 0.05 was considered statistically significant.

## 3. Results

**Figure 1:**
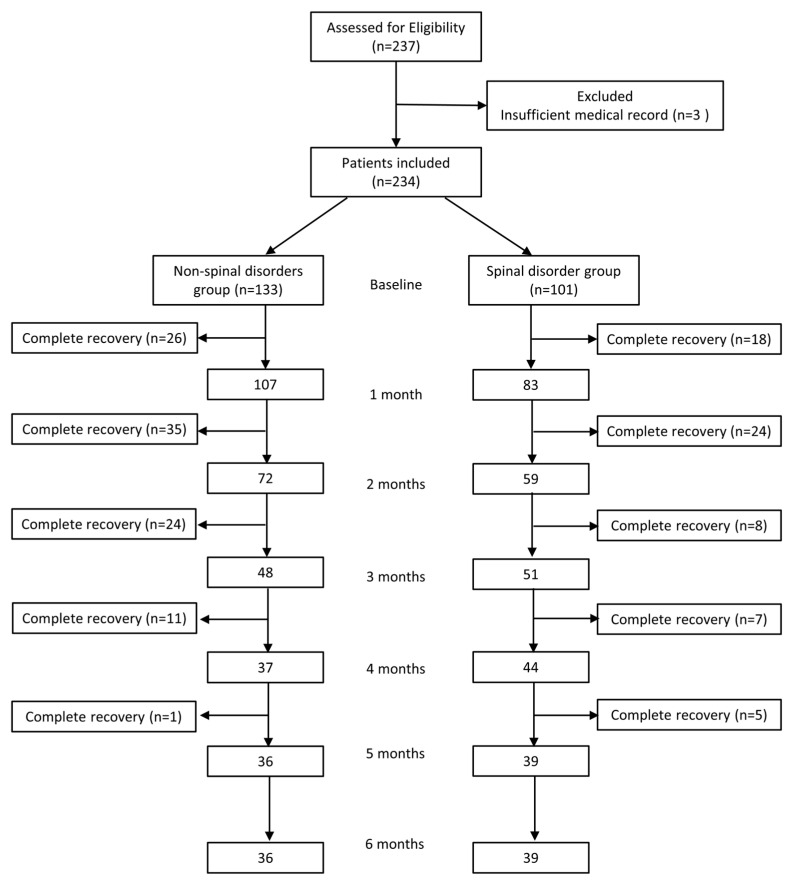
Flow diagram indicating patient progress through the study.

During the study period, 237 patients were enrolled, of whom 234 satisfied all inclusion criteria, and 3 were disqualified due to exclusion criteria. The patients who reached an NRS score of zero and no longer required conservative treatment were considered to have achieved complete recovery. Ultimately, the non-spinal disorder group included 133 patients, and the spinal disorder group included 101 patients (Figure 1). Table 1 presents patient demographic information and characteristics. No significant differences were observed between both groups in age, sex, number of injections, side of symptoms, involvement of a dermatome, NRS score, pain duration, or underlying disease.

The types of spinal disorders included spinal stenosis, herniated nucleus pulposus, compression fracture, and spondylosis (Table 2).

**Figure 2:**
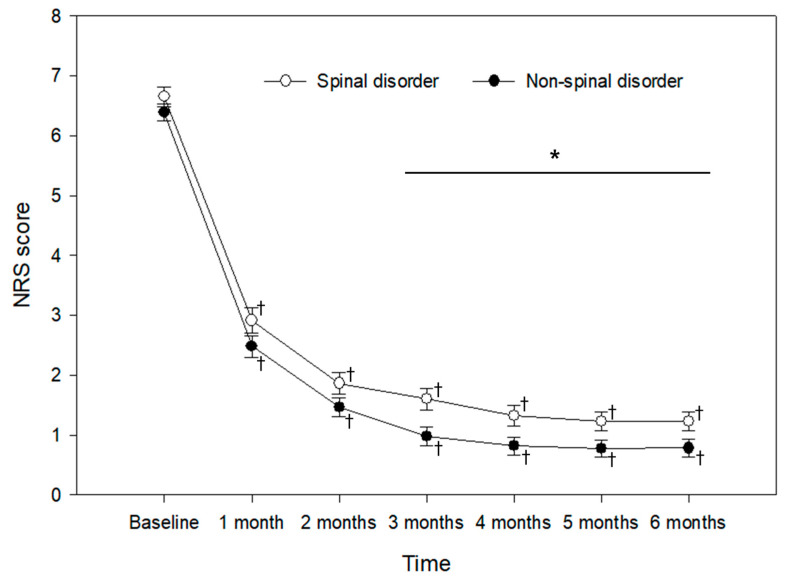
Pain intensity of patients with remaining ZAP at different time points. Values are presented as mean ± standard error for NRS. * *p* < 0.05: comparison of differences between the groups; † *p* < 0.05: comparison of each variable at specific time points with baseline. ZAP, Zoster-associated pain; NRS, numeric rating scale.

Both groups demonstrated significant changes in NRS scores over time compared to baseline values. The NRS values were higher in the spinal disorder group at 3–6 months than in the non-spinal disorder group (*p* < 0.05; Figure 2). Table 3 reveals that there were no noteworthy differences between both groups in the proportion of clinically significant PHN.

Anticonvulsant doses and MEDD were not significantly different between the non-spinal and spinal disorder groups (Table 4 and Table 5).

**Figure 3:**
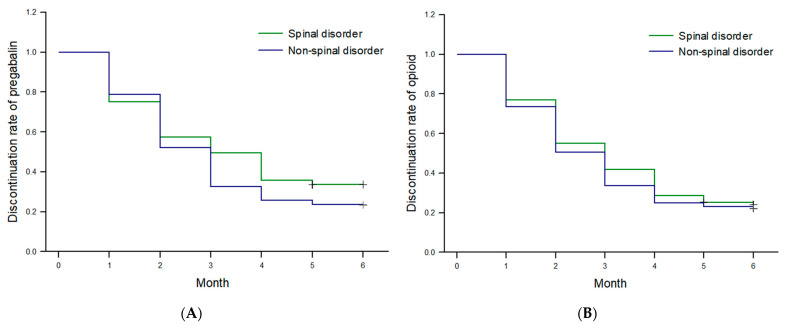
Kaplan–Meier curve: (**A**) Discontinuation rate of pregabalin. (**B**) Discontinuation rate of MEDD. There was no significant difference observed between the groups in either of the two graphs.

There was no significant difference in the rate of discontinuing anticonvulsants between both groups at any time point (Figure 3).

## 4. Discussion

In this study, we attempted to determine whether the degree of ZAP is greater and whether the incidence of PHN is higher in individuals with existing spinal disorders. Patients with existing spinal disorders had significantly higher NRS scores at 3–6 months; however, their likelihood of developing PHN did not increase. Moreover, there were no significant differences in the drug dosage or duration of drug maintenance between both groups. Regarding the increased severity of pain when the two conditions coexist, we speculated two potential causes. First, we considered the applicability of the concept of “double crush syndrome (DCS)” by acknowledging its similarity to the etiology of PHN following HZ. Second, we posited that the presence of spinal disorders might result in reduced physical activity, contributing to higher average pain intensity 3–6 months after HZ onset. Although the presence of spinal disorders may not directly influence the incidence of PHN, it seemed to play a role in impacting the overall pain experience following the occurrence of HZ.

DCS is a condition in which multiple nerves are compressed at two or more sites, causing symptoms at the same nerve pathway that may exhibit a synergistic effect [25,26,27]. Although this concept is not directly related to the current study, it suggests that pain may synergistically increase when multiple disorders coexist at the same sensory level [25,26,27]. The mechanisms that are the most plausible causes of DCS, including axonal transport, ion channel changes, inflammation of the dorsal root ganglion (DRG), and central sensitization resulting from nerve compression caused by pre-existing spinal disorders, are also strongly associated with the mechanisms underlying PHN development [27,28,29,30,31,32,33,34,35,36,37].

PHN is a noteworthy clinical problem that can last for several years and has an adverse impact on the patient’s quality of life in all four health domains—physical, psychological, functional, and social [5,8,38]. Despite numerous studies, the pathophysiology of PHN remains unclear. However, PHN is also closely related to the aforementioned mechanisms underlying the development of DCS, including axonal transport, ion channel changes, inflammation of the DRG, and central sensitization [28,29,30,31,32,33,34,35,36,37]. The mechanism of axonal transport, which carries newly produced viral particles along the central and distal axons of sensory neurons, leads to widespread necrosis and cell death in the skin, nerve, root, and ganglion and can affect the development of PHN [28,33]. Several studies suggest that varicella-zoster virus (VZV) may contribute to the pathogenesis of PHN by directly affecting voltage-gated sodium ion channels [30,31]. Nerve damage is often associated with the dysfunction of ion channels, including voltage-gated calcium ion channels [30,37]. The dysfunction of ion channels can lead to abnormal ion signals in cells, which may contribute to the development of pain and persistent symptoms in PHN [30,31,37]. During VZV infection, the reactivated virus can cause an inflammatory immune response, leading to necrosis and cell death in the nerve root and ganglion [29,35,36]. The damage to the nerve root and ganglion during a VZV infection can result in a lowered threshold for painful stimuli, hyperalgesia, and allodynia, ultimately contributing to the development of PHN [29,35,36]. Central sensitization is one of the major causes proposed for the development of PHN. VZV-induced nerve inflammation can compromise the dorsal horns and impair the descending inhibitory pain pathways, which may ultimately lead to central sensitization [32,33,34,36].

Individuals aged ≥60 years have a higher risk of developing HZ and PHN, with studies indicating that the incidence of PHN increases with age [10,11,14]. Furthermore, the number of individuals affected by PHN and the related medical expenses steadily increase every year. This trend could be linked to an increasing older adult population and extended life expectancy [4]. Each year, over 20% of individuals aged ≥65 years experience chronic low back pain, with back pain in older adults commonly attributed to the degenerative changes that are prevalent in this age group and escalate with advancing years [39]. The aging spine is affected by the conditions included in this study, such as “spinal stenosis” and “disc disease”, and the degenerative process of these spinal disorders is multifaceted [9,19,20,21,39,40]. Spinal osteoarthritis and disc degeneration, both common spinal conditions that result in neck and back pain, particularly in older individuals, also become more prevalent with age [9,21]. Among spinal conditions, spinal stenosis is considered the most debilitating and is typically observed in older patients. The incidence of symptomatic lumbar spinal stenosis is estimated to be approximately 47% in individuals aged >60 years [9,20]. Individuals aged >75 years with a history of trauma, osteoporosis, severe pain, or thoracic pain have a high risk of a positive diagnosis of vertebral compression fractures [9,19]. Spinal disorders are linked to diminished physical performance measures, subsequently elevating the likelihood of health issues, limitations in daily activities, and incidents of falling [9,19,20,21,39,40]. Functional impairments resulting from these spinal conditions are often assessed using scores such as the SF-12 physical component score, which has been used in several studies to measure the extent of spinal impairment [41,42].

Previous studies have reported that physical functional impairment may impact the occurrence of PHN following HZ [38,43,44,45,46]. Bouhassira et al. [43] conducted univariate analyses to identify significant factors related to PHN 3 months after onset. Taking these factors into consideration, they performed a multivariable analysis and found that a low PCS score was an independent predictor for PHN. Drolet et al. [44] also investigated the influence of socio-demographic and general health characteristics on the risk of developing PHN. They found that older age and pre-existing mobility prior to HZ infection significantly increased the risk of PHN. Similarly, Kawai et al. [38] reported that in their multivariable regression model, older age (60–69 vs. 50–59 years), greater severity of pain at rash onset, employment status, mobility problems at enrollment, and pain affecting interpersonal relationships were significantly associated with the development of PHN.

The precise associations between spinal disorders and the incidence of HZ, as well as the severity of ZAP and the increase in PHN rates, have not been definitively established yet. In a study conducted by Alpantaki et al. [47], it was proposed that the detection of herpes virus DNA in intervertebral disc samples from patients with lumbar disc herniation suggests the potential involvement of herpes viruses in the development of degenerative disc disease. Multiple studies have sought to elucidate the relationship between viral infections and apoptosis [48,49]. The presence of viral DNA in intervertebral discs might be linked to the migration of cells containing viral genetic material, such as macrophages, during childhood when the disc environment is still well vascularized [50,51]. Furthermore, the activation of the herpes simplex virus in neuronal axons by inflammatory cytokines and antidromal migration could potentially influence the disc space condition [52].

There are reports indicating that stimuli such as trauma and surgery can trigger the onset of HZ. Additionally, there are reports suggesting that spinal disorders themselves can induce the manifestation of HZ [1,53,54,55,56]. The research suggests that trauma at a specific site increases the risk of zoster in that area, implying that nerve stimulation from trauma might reactivate latent virus in the DRG [1,53,54]. In addition, trauma may disrupt local cutaneous immunity or stimulate local sensory nerves, leading to VZV reactivation and the subsequent development of HZ [53,54]. Both possibilities could explain the rapid development of HZ following trauma and may not exclude each other. There have been a few reported cases of HZ-mediated radiculitis after a major surgical intervention, when the body is under significant stress or relatively immunocompromised [55,56]. Several studies have reported that disc degeneration, including herniation, could increase the risk of HZ occurrence [57,58,59]. Hata et al. [58] found, through multivariate analysis, that disc herniation is associated with an increased incidence of HZ. Additionally, Dhillon et al. [57] suggested that a foraminal disc protrusion may impinge on the DRG located in the neural foramen, leading to radicular pain and potentially triggering HZ reactivation. In a study conducted by Ke et al. [59], it was suggested that sciatica caused by conditions including herniated nucleus pulposus could potentially increase the risk of HZ occurrence. They explored whether sciatica could act as a stressor leading to HZ development. The study speculated that stress and pain could lead to changes in the body’s perceptual and stress systems, resulting in abnormal output patterns from the neuromatrix. These mechanisms are believed to be linked to decreased HZ-specific cellular immunity, thereby increasing the risk of HZ in patients with sciatica. Although the exact association and causality between the two conditions have not been definitively established, it cannot be ruled out that the presence of spinal disorders may potentially influence the occurrence and severity of HZ and PHN.

ZAP can be mistaken for symptoms related to other neuromuscular or spinal disorders since differentiating between neuropathic pain caused by spinal disorders and PHN is difficult, particularly before shingles blisters develop [56,60,61,62]. In addition, distinguishing between segmental zoster paresis and a combination of spinal radiculopathy and motor neuropathy can be challenging [56,60,61,62]. Both conditions share similarities in their pharmacological and interventional treatment options [4,15,16,63]. Anticonvulsants and opioids, such as pregabalin and gabapentin, are usually used as medical treatments for neuropathy caused by spinal disorders and ZAP [15,63], and conservative treatments such as SNRB are similarly applied [4,16]. In this study, both groups underwent the same SNRB procedure. While SNRB could potentially impact the development of PHN and improve the condition of spinal disorders, we were unable to establish a definitive causal relationship due to the nature of the study design. Nevertheless, there was no significant difference in the number of SNRBs performed between the two groups. Although the concurrent presence of spinal disorders and HZ did not increase the risk of PHN in this study, individuals with spinal disorders present from the third month after HZ exhibited significantly higher levels of pain. These results suggest that when spinal disorders and HZ coexist at the same nerve level, prioritizing pain management is crucial for the effective treatment of ZAP. In the future, more extensive investigations, such as prospective randomized trials or meta-analyses, will be necessary to evaluate the influence of comorbidities, including not only spinal disorders but also other conditions coexisting with zoster infection, on ZAP and PHN.

This study has some limitations. First, its retrospective nature might have led to variability among study participants. Nevertheless, heterogeneity was reduced by standardizing patient demographics, clinical variables, and imaging findings before treatment and at each subsequent follow-up visit. Second, while checking for spinal disorders, only the existing pain score was reviewed in the records, and precise details such as the date of diagnosis, physical functional scores at the initial visit, and type of treatment administered were not verified. In addition, despite the absence of significant differences in the initial medication dosages or pain scores during the first visit, the assessment of the patients’ spinal disorders remains inadequate due to the lack of pre-visit data on medication doses and pain levels. Third, although there may exist patients without symptoms despite having imaging findings related to spinal disorders, this study specifically targeted patients presenting with pain during their initial visit. Therefore, all patients showing imaging evidence of spinal disorders were inevitably included in the spinal disorder group.

## 5. Conclusions

In conclusion, our study demonstrates that although the incidence of PHN was not significantly higher, the presence of spinal disorders at the same sensory level resulted in a significant increase in ZAP during the 3–6 months after HZ infection. Therefore, when both conditions coexist at the same sensory level, the pain intensity tends to be higher than when they are not present together, indicating the need for meticulous pain management. Conducted retrospectively, this study focuses on a specific ethnic group within a single country, despite having limitations such as a relatively small sample size. Nevertheless, this study demonstrates that the presence of concurrent conditions can potentially impact ZAP, suggesting a preliminary platform for exploring complex interactions between HZ and other conditions. Moving forward, large multicenter investigations will be essential to comprehend the intricate relationships among these diseases with precision.

## Figures and Tables

**Table 1:** General patient characteristics.

	Non-Spinal Disorder (*n* = 133)	Spinal Disorder (*n* = 101)	*p* Value
Age (year)	65.196 ± 13.579	68.327 ± 13.895	0.085
Sex			0.064
Female	92 (69.2%)	58 (57.4%)	
Male	41 (30.8%)	43 (42.6%)	
Number of SNRBs	3.301 (2.50)	3.030 (2.0)	0.365
Side of symptom			0.249
Right	61 (45.9%)	54 (53.5%)	
Left	72 (54.1%)	47 (46.5%)	
Involved dermatome			0.100
Cervical	23 (17.3%)	13 (12.9%)	
Thoracic	86 (64.7%)	58 (57.4%)	
Lumbar	21 (15.8%)	29 (28.7%)	
Sacral	3 (2.3%)	1 (1.0%)	
NRS	6.391 ± 1.651	6.653 ± 1.746	0.241
Pain duration (month)	20.541 ± 14.696	23.089 ± 19.807	0.260
Underlying disease			0.340
Hypertension	33 (24.8%)	33 (32.7%)	
Diabetes mellitus	11 (8.3%)	7 (6.9%)	
Hypertension and Diabetes mellitus	5 (3.8%)	6 (5.9%)	
None	84 (63.2%)	55 (54.5%)	

Values are presented as mean ± standard deviation, median (interquartile range), or number of patients (%). SNRB, selective nerve root block; NRS, numeric rating scale.

**Table 2:** Types of spinal disorders.

Type of Disease	
Spinal stenosis	16 (15.8%)
Herniated nucleus pulposus	28 (27.7%)
Compression fracture	25 (24.7%)
Spondylosis	32 (31.7%)

Values are presented as number of patients (%).

**Table 3:** Rate of PHN at follow-up.

	Proportion of All Patients		
Time	Non-Spinal Disorder (*n* = 133)	Spinal Disorder (*n* = 101)	*p* Value
3 months	18.8% (25/133)	26.7% (27/101)	0.148
4 months	16.5% (22/133)	23.8% (24/101)	0.169
5 months	16.5% (22/133)	22.8% (23/101)	0.231
6 months	16.5% (22/133)	22.8% (23/101)	0.231

Values are presented as number of patients (%). PHN, postherpetic neuralgia.

**Table 4:** Anticonvulsant (pregabalin) doses at follow-up.

Time	Non-Spinal Disorder (*n* = 133)	Spine Disorder (*n* = 101)	*p* Value
Baseline	183.271 ± 85.165 (*n* = 133)	196.634 ± 86.661 (*n* = 101)	0.297
1 month	175.278 ± 86.459 (*n* = 108)	174.405 ± 96.084 (*n* = 84)	0.830
2 months	170.000 ± 94.225 (*n* = 77)	187.705 ± 90.100 (*n* = 61)	0.330
3 months	168.8000 ± 102.432 (*n* = 50)	192.115 ± 87.567 (*n* = 52)	0.295
4 months	186.923 ± 100.476 (*n* = 39)	166.279 ± 106.749 (*n* = 43)	0.317
5 months	172.105 ± 106.318 (*n* = 38)	157.895 ± 107.349 (*n* = 38)	0.434
6 months	177.368 ± 110.078 (*n* = 38)	159.211 ± 106.926 (*n* = 38)	0.352

Values are presented as mean ± standard deviation.

**Table 5:** Opioid (MEDD) doses at follow-up.

Time	Non-Spine Disorder (*n* = 133)	Spine Disorder (*n* = 101)	*p* Value
Baseline	13.847 ± 13.576 (*n* = 133)	19.179 ± 18.484 (*n* = 101)	0.081
1 month	11.957 ± 13.646 (*n* = 108)	17.187 ± 18.749 (*n* = 83)	0.059
2 months	11.585 ± 13.156 (*n* = 73)	18.100 ± 19.789 (*n* = 61)	0.086
3 months	9.149 ± 10.095 (*n* = 50)	18.064 ± 21.320 (*n* = 52)	0.349
4 months	11.698 ± 17.195 (*n* = 39)	17.426 ± 22.759 (*n* = 43)	0.992
5 months	11.709 ± 17.442 (*n* = 38)	17.334 ± 23.093 (*n* = 38)	0.996
6 months	11.117 ± 17.643 (*n* = 38)	17.597 ± 23.384 (*n* = 38)	0.744

Values are presented as mean ± standard deviation. MEDD, morphine equivalent daily dose.

## Data Availability

Not applicable.
